# Effects of Reciprocal Ia Inhibition on Contraction Intensity of Co-contraction

**DOI:** 10.3389/fnhum.2018.00527

**Published:** 2019-01-11

**Authors:** Ryo Hirabayashi, Mutsuaki Edama, Sho Kojima, Masatoshi Nakamura, Wataru Ito, Emi Nakamura, Takanori Kikumoto, Hideaki Onishi

**Affiliations:** Institute for Human Movement and Medical Sciences, Niigata University of Health and Welfare, Niigata, Japan

**Keywords:** H-reflex, M wave, electromyograph, joint movement, co-contraction

## Abstract

**Introduction:** Excessive co-contraction interferes with smooth joint movement. One mechanism is the failure of reciprocal inhibition against antagonists during joint movement. Reciprocal inhibition has been investigated using joint torque as an index of intensity during co-contraction. However, contraction intensity as an index of co-contraction intensity has not been investigated. In this study, we aimed to evaluate the influence of changes in contraction intensity during co-contraction on reciprocal inhibition.

**Methods:** We established eight stimulus conditions in 20 healthy adult males to investigate the influence of changes in contraction intensity during co-contraction on reciprocal inhibition. These stimulus conditions comprised a conditioning stimulus-test stimulation interval (C–T interval) of -2, 0, 1, 2, 3, 4, or 5 ms plus a test stimulus without a conditioning stimulus (single). Co-contraction of the tibialis anterior and soleus muscles at the same as contraction intensity was examined at rest and at 5, 15, and 30% maximal voluntary contraction (MVC).

**Results:** At 5 and 15% MVC in the co-contraction task, the H-reflex amplitude was significantly decreased compared with single stimulation at a 2-ms C–T interval. At 30% MVC, there was no significant difference compared with single stimulation at a 2-ms C–T interval. At a 5-ms C–T interval, the H-reflex amplitude at 30% MVC was significantly reduced compared with that at rest.

**Discussion:** The findings indicated that during co-contraction, reciprocal Ia inhibition worked at 5 and 15% MVC. Contrary inhibition of reciprocal Ia inhibition did not apparently work at 30% MVC, and presynaptic inhibition (D1 inhibition) might work.

## Introduction

In many upper motor neuron disorders including spastic diseases, cerebellar ataxia, Parkinson’s disease, and spinal cord injury, when movement of only an agonist is required (at the time of reciprocal inhibition exercise), a collapse of the reciprocal inhibition mechanism against the antagonist causes excessive co-contraction. Thus, reciprocal inhibition does not work on the antagonist, impairing smooth joint movement (Hayashi et al., 1988; Kagamihara et al., 1993; Kagamihara and Tanaka, 1996). Such excessive co-contraction occurs in pathological states, but the amount of co-contraction muscle activity is also known to increase with age (Morita et al., 1995, 2000; Hortobagyi and Devita, 2006; Hortobagyi et al., 2009; Baudry et al., 2010; Nagai et al., 2011). In addition, excessive co-contraction in athletes may interfere with joint movement and degrade performance requiring agility (Blackwell and Cole, 1994). Factors involved in excessive co-contraction include decreased reciprocal Ia inhibition from agonist Ia fibers (Mizuno et al., 1971; Nielsen and Kagamihara, 1992; Crone et al., 1994; Okuma et al., 2002), decreased D1 inhibition (Mizuno et al., 1971; Tanaka, 1974; Crone and Nielsen, 1989; Faist et al., 1994; Nielsen et al., 1995), and antagonist-prompting input (Crone et al., 2000). Collectively, these findings indicate that many factors can cause excessive co-contraction.

A previous study on reciprocal inhibition during co-contraction of the tibialis anterior (TA) and soleus (Sol) muscles in healthy men reported that the amount of reciprocal Ia inhibition was further decreased during co-contraction than at rest (Nielsen and Kagamihara, 1992). In that study, muscle force (joint torque) was used as an index of co-contraction intensity (Nielsen and Kagamihara, 1992). Several other studies on reciprocal inhibition during isometric contraction of plantar- and dorsiflexion also evaluated co-contraction intensity as joint torque (Nielsen and Kagamihara, 1992; Nielsen et al., 1992, 1994; Morita et al., 2001). Such studies found that, because several muscles are involved in plantar- and dorsiflexion during co-contraction, the amounts of TA and Sol muscle activity (contraction intensity) are different. However, the contraction intensities of TA and Sol are unknown. The H-reflex and the amount of reciprocal Ia inhibition may vary (Nielsen et al., 1994; Morita et al., 2001); hence, the muscle activity should be determined during co-contraction. In consideration of the proportion of exercise units recruited as the output of each muscle, the present study focused on the amount of muscle activity during co-contraction, warranting the investigation of the reciprocal Ia inhibition in co-contraction with the same amount of muscle activity and not joint torque. It is thus expected that the contraction intensities of TA and Sol are identical and that the contraction intensity should change during co-contraction to elucidate reciprocal inhibition.

This study thus aimed to clarify the influence of changes in contraction intensity during co-contraction on reciprocal inhibition. Other researchers (e.g., Nielsen and Kagamihara, 1992) have reported that reciprocal Ia inhibition decreases during co-contraction compared to rest, but whether reciprocal Ia inhibition disappears is unclear. As the amount of reciprocal Ia inhibition depends on contraction intensity, our working hypothesis was that during co-contraction, at the same contraction intensity of TA and Sol, reciprocal Ia inhibition should persist when contraction intensity is low and decrease as it increases.

## Materials and Methods

### Study Participants

Twenty healthy adult males (age, 21.3 ± 1.0 years; height, 171.7 ± 5.5 cm; body weight, 61.6 ± 4.6 kg) provided written informed consent to participate in this study. The study was performed in accordance with the Declaration of Helsinki and approved by the Ethics Committee at Niigata University of Health and Welfare.

### Measurements of Limb Positions

**FIGURE 1:**
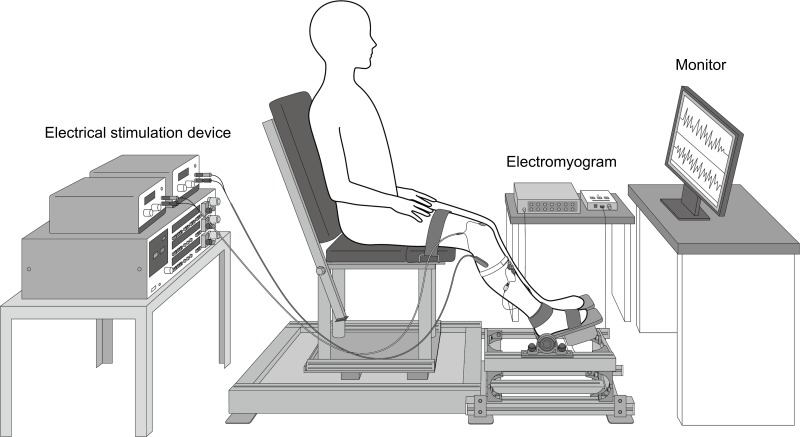
Limb position for measurements. We measured the right hip flexion (90°), right knee joint (120°), and ankle joint (90°). We stimulated the dominant deep peroneal nerve of the tibialis anterior muscle (TA). The test stimulus was applied to the dominant tibial nerve of the soleus muscle (Sol). Electromyography electrodes were affixed to the belly of the TA and Sol muscles.

The right lower limb position was measured at the hip (90°), knee (120°), and ankle (90°) joints. The ankle was immobilized using a foot plate (TAKEI SCIENTIFIC INSTRUMENTS, Niigata, Japan) (Figure 1). Prior to the start of the experiment, participants practiced contracting the TA and Sol muscles without moving the ankle.

### Electromyography

The distance between the Ag/AgCl electrodes (Blue Sensor, METS, Tokyo, Japan) of the surface electromyogram was set to 20 mm. The electrodes were placed on the TA and Sol muscles according to SENIAM (Hermens et al., 2000). The ground electrode was placed between the electrical stimulation electrode and the surface electromyogram electrode. Electromyographic activity was filtered at a band-pass filter of 10–1,000 Hz and amplified 100× (FA-DL-720-140; 4Assist, Tokyo, Japan) before being digitally stored (10 kHz sampling rate) on a personal computer for offline analysis. Analysis was performed using PowerLab 8/30 (AD Instruments, Colorado Springs, CO, United States) and LabChart 7 (AD Instruments).

### Electrical Stimulation

Muscles were stimulated for 1 ms (rectangular wave) using a SEN-8203 electrical stimulation device (Nihon Kohden, Tokyo, Japan) via a SS-104J isolator (Nihon Kohden). The tibial nerve was selectively stimulated in a monopolar fashion to induce the Sol H-reflex and M waves. The anode and cathode were located on the upper patella and the popliteal area, respectively, for the test stimulus. M waves were induced in the TA using bipolar stimulation, and a conditioning stimulus was applied along the deep peroneal nerve below the fibula head (Mizuno et al., 1971; Crone et al., 1987; Nielsen and Kagamihara, 1992).

### Measurement of Reciprocal Inhibition

Reciprocal inhibition was measured as described previously (Mizuno et al., 1971; Crone et al., 1987; Nielsen and Kagamihara, 1992). A test stimulus was applied to the dominant (tibial) nerve of the Sol after a conditioning stimulus was delivered to the dominant (deep peroneal) nerve of the TA, and then the Sol H-reflex amplitude value was recorded. Condition stimulation preceding the deep peroneal nerve suppresses the excitability of Sol’s spinal cord anterior horn cells via inhibitory interneurons. Therefore, when the test stimulus is later applied to the tibial nerve, the Sol H-reflex amplitude value decreases. The eight stimulation conditions comprised a conditioning stimulus–test stimulation interval (C–T interval) of -2, 0, 1, 2, 3, 4, or 5 ms plus a test stimulus without a conditioning stimulus (single). A set of 8 random conditions was repeated 10 times, for a total of 80 stimuli, and delivered at a frequency of 0.3 Hz. There was a 1-min period of rest between sets. The intensity of the conditioning stimulus was set to the M wave threshold of the TA. Because the amount of reciprocal Ia inhibition varies with the size of the H-reflex (Crone et al., 1990), the intensity of the test stimulus was set to elicit H-reflex, reaching 15–25% of the maximum amplitude of the Sol M wave (Mmax).

### Co-contraction Task

The contraction intensities of TA and Sol were determined by measuring the maximal voluntary contraction (MVC). MVC was performed for plantar- and dorsiflexion with the thighs and feet fixed as shown in Figure 1. Co-contraction of TA and Sol at the same contraction intensity was examined at rest and at 5, 15, and 30% MVC, with rest intervals of at least 3 min. The maximum intensity of co-contraction was 30% MVC, which permitted co-contraction without change to the joint angle. A monitor positioned in front of the participants displayed feedback regarding the amount of muscle activity. During co-contraction, the foot was immobilized with a plate to prevent any change in the angle of the ankle. Prior to the experiments, participants practiced performing muscle activity without moving the ankle joint.

### Experimental Protocol

**FIGURE 2:**
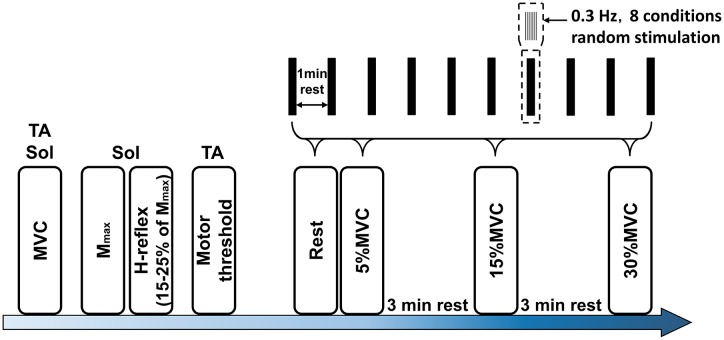
Experimental protocol. Mmax, maximum motor response; MVC, maximal voluntary contraction; Sol, soleus; TA, tibialis anterior muscle.

The experimental procedure is shown in Figure 2. The MVC of TA and Sol was measured, and 5, 15, and 30% MVC of each muscle was then calculated. For the soleus H-reflex, the stimulus intensity during the experiment was adjusted to provide an H-reflex of 15–25% of this value both at rest and during movement (Nielsen and Kagamihara, 1992). Next, the conditioning stimulation to the TA deep peroneal nerve and the threshold intensity of the M wave were established. The eight random stimulation conditions comprised the single interval and -2, 0, 1, 2, 3, 4, and 5-ms intervals at a frequency of 0.3 Hz. Rest intervals of at least 3 min were allowed between the 80 (8 conditions × 10 sets) stimulation sets.

### Statistical Processing

Peak-to-peak Sol H-reflex amplitudes were calculated as mean ± standard error (SE). The reciprocal inhibition of each co-contraction task was calculated as a percentage by dividing the Sol H-reflex amplitude by the Mmax amplitude ([Sol H-reflex amplitude/Mmax amplitude] × 100). To compare the amount of reciprocal inhibition between simultaneous contraction tasks under the seven stimulation conditions (excluding the single condition), the H-reflex amplitude for the conditioning stimulus was divided by the H-reflex amplitude of only the test stimulus and expressed as a percentage ([Conditioned H-reflex amplitude/Test H-reflex amplitude] × 100). Values are expressed as mean ± standard deviation. Iterative values were generated using two-way repeated-measures analysis of variance of the factors, co-contraction task, and stimulation conditions. *Post hoc* Tukey–Kramer multiple comparison tests were then performed. The seven stimulation conditions were compared with the single condition using paired *t*-tests with the Bonferroni correction for multiple comparisons. Co-contraction tasks under each stimulation condition were compared using two-way repeated-measures ANOVA with the factors, co-contraction task, and stimulation condition, followed by *post hoc* Tukey–Kramer multiple comparison tests. Statistical significance was set at *p* < 0.05.

## Results

### Test H-Reflex Amplitude for Each Co-contraction Task (Table 1)

**Table 1:** Test H-reflex amplitudes for co-contraction tasks.

	Rest	5% MVC	15% MVC	30% MVC
Test H-reflex amplitude (% of Mmax)	16.63 ± 1.03	16.94 ± 1.37	17.58 ± 1.44	18.37 ± 2.06


*Data are shown as means ± SE.*

In the two-way repeated measures ANOVA of the co-contraction task and stimulation conditions, there was a main effect of the stimulus condition [*F*(7,133) = 36.271, *p* < 0.001, partial η^2^ = 0.656], but not of the co-contraction task [*F*(3,57) = 0.327, *p* = 0.806, partial η^2^ = 0.017]. In addition, the stimulation condition and the co-contraction task interacted significantly [*F*(21,399) = 6.261, *p* < 0.001, partial η^2^ = 0.248]. Analyses of the single Sol H-reflex amplitudes during each co-contraction task and multiple comparisons did not identify any significant differences. These findings confirmed that changes in the Sol H-reflex amplitude in response to conditioning stimuli do not depend on the test stimulus intensity.

### H-Reflex Amplitude Between Stimulation Conditions (Figure 3)

**FIGURE 3:**
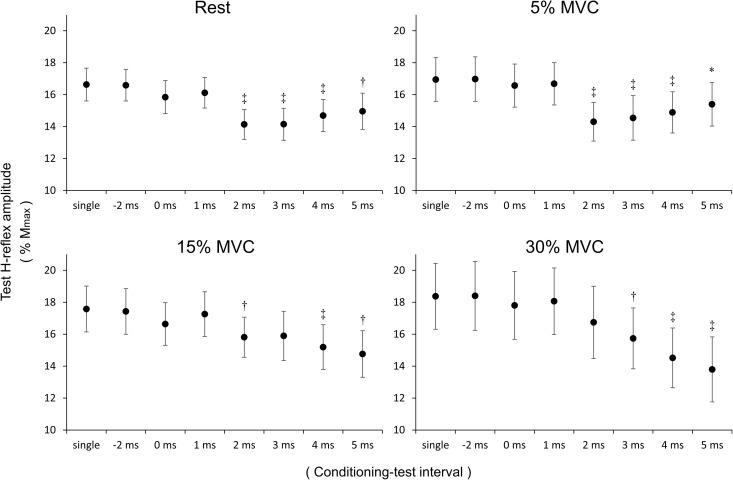
H-reflex amplitude value between stimulation conditions. The vertical axis represents H-reflex/Mmax × 100 for the four co-contraction tasks (rest, 5% MVC, 15% MVC, 30% MVC). Values are mean ± standard error. Data were statistically analyzed by comparing the H-reflex amplitude value of the single condition (divided by Mmax value) vs. the H-reflex amplitude value (divided by Mmax value) of each of the seven conditions (–2, 0, 1, 2, 3, 4, and 5 ms). MVC, maximal voluntary contraction; ^∗^*p* < 0.05, ^†^*p* < 0.01, ^‡^*p* < 0.001.

The seven stimulation conditions were compared with the single condition using paired *t*-tests and the Bonferroni correction. Compared with the single condition, the rest H-reflex amplitude was significantly reduced at C–T intervals of 2 (*p* < 0.001), 3 (*p* < 0.001), 4 (*p* < 0.001), and 5 (*p* = 0.001) ms. Similarly, at 5% MVC, the H-reflex amplitude was significantly reduced at C–T intervals of 2 (*p* < 0.001), 3 (*p* < 0.001), 4 (*p* < 0.001), and 5 (*p* = 0.038) ms compared with the single condition. At 15% MVC, the H-reflex amplitude was significantly reduced at C–T intervals of 2 (*p* = 0.002), 4 (*p* < 0.001), and 5 (*p* = 0.001) ms compared with the single condition. At 30% MVC, the H-reflex amplitude was significantly reduced at C–T intervals of 3 (*p* = 0.001), 4 (*p* < 0.001), and 5 (*p* < 0.001) ms compared with the single condition.

### H-Reflex Amplitude Between Co-contraction Tasks

**FIGURE 4:**
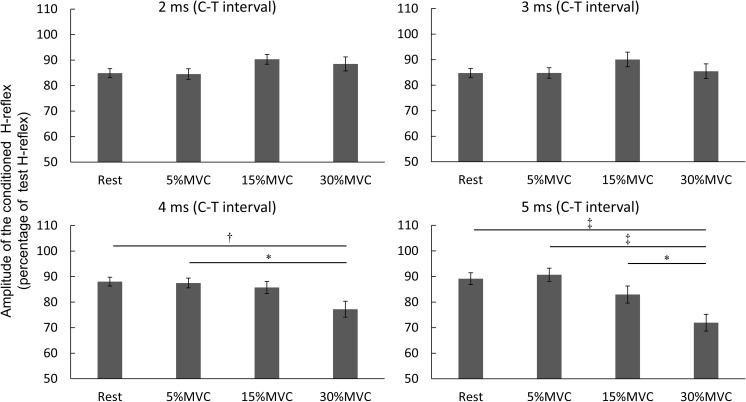
H-reflex amplitude between co-contraction tasks. The vertical axis is the amplitude of conditioning H-reflex/Amplitude of test H-reflex × 100 of the four co-contraction tasks (rest, 5, 15, and 30% MVC). Values are mean ± standard deviation. Two factors comprising the co-contraction task and stimulation condition were iteratively measured using two-way repeated-measures analysis of variance (ANOVA) and the Tukey–Kramer *post hoc* test for multiple comparisons. MVC, maximal voluntary contraction; ^∗^*p* < 0.05, ^†^*p* < 0.01, ^‡^*p* < 0.001.

Figure 4 depicts the comparisons of the co-contraction tasks at each stimulation conditions. Two-way repeated-measures ANOVA revealed the main effect [*F*(6,114) = 39.024, *p* < 0.001, partial η^2^ = 0.673] of each stimulation condition but not that of the co-contraction task [*F*(3,57) = 1.505, *p* = 0.223, partial η^2^ = 0.073]. Additionally, the stimulation condition and co-contraction task interacted significantly [*F*(18,342) = 9.027, *p* < 0.001, partial η^2^ = 0.322]. Multiple comparison tests, followed by *post hoc* analyses, did not identify any significant differences between co-contraction tasks at C–T intervals of -2, 0, 1, or 2 ms. In the co-contraction task at 30% MVC at a C–T interval of 4 ms, the H-reflex amplitude was significantly reduced compared with that at rest (*p* = 0.009) and at 5% MVC (*p* = 0.014). In the co-contraction task at 30% MVC with a C–T interval of 5 ms, the H-reflex amplitude was significantly reduced compared with that at rest (*p* < 0.001) as well as that at 5% (*p* < 0.001) and 15% MVC (*p* = 0.045).

## Discussion

The main findings of the present study are that reciprocal Ia inhibition occurred at 5 and 15% MVC due to changes in contraction intensity during co-contraction at the same intensity between the TA and Sol muscles. In addition, we demonstrated that the amount of reciprocal Ia inhibition during co-contraction of 5 and 15% MVC was the same as that at rest. Reciprocal Ia inhibition was not evident at 30% MVC, suggesting D1 inhibition.

Our findings at rest were similar to those of a previous study (Nielsen and Kagamihara, 1992), which showed that the Sol H-reflex amplitude decreased significantly at a C–T interval of 2 ms. Furthermore, suppression at a C–T interval of 2 ms is consistent with findings from experimental animal studies (Baldissera et al., 1981) and is thought to be due to two synaptic reciprocal Ia inhibitions (Okuma and Lee, 1996). The extent of inhibition was large at a C–T interval of 2 ms in the present study, indicating that the excitability of Sol alpha motoneurons was attenuated by two synaptic reciprocal Ia inhibitions, as described previously.

During co-contraction at 5 and 15% MVC, the Sol H-reflex amplitude was significantly decreased at a C–T interval of 2 ms compared with the single condition. These findings are similar to our hypothesis, as reciprocal inhibition differed from previous findings, and the extents of reciprocal Ia inhibition at 5 and 15% MVC were the same as those at rest. Previous studies have investigated changes in the amounts of reciprocal Ia inhibition during co-contraction at the same joint torque during plantar- and dorsiflexion (Nielsen and Kagamihara, 1992). By contrast, our study examined changes in reciprocal Ia inhibition during TA and Sol co-contraction at the same as contraction intensity. A previous study on the amount of reciprocal Ia inhibition and the changes in the strength of joint torque found that reciprocal Ia inhibition constantly acted on Sol independently of muscle strength during isometric contraction of dorsiflexion. When bending the ankle joint, reciprocal Ia inhibition was shown to depend on the strength of plantarflexion and decrease with increasing Sol muscle strength (Nielsen and Kagamihara, 1992). Here, we considered that the amount of reciprocal Ia inhibition of TA and Sol at the same co-contraction intensity might depend on the contraction intensity of Sol. In co-contraction with the same TA and Sol muscle activities in this study, it may be a low force level at 15% MVC or less. One previous study (Nielsen and Kagamihara, 1992) showed that the plantarflexion torque revealed reciprocal Ia inhibition of up to 6 Nm, so we also suggest that the reciprocal Ia inhibition could be recognized in co-contraction <15% MVC. Excessive co-contraction reduces exercise performance of joint movement, whereas moderate co-contraction reportedly increases ligament function, improves joint stability, equalizes pressure distribution on the joint surface, and prevents bone displacement (Crone and Nielsen, 1994). Thus, antagonist muscle activity should be minimized to efficiently contract an agonist muscle to produce torque during joint movement (Hortobagyi and Devita, 2006; Loram et al., 2014). In terms of co-contraction intensity, 5 and 15% MVC increased the amount of reciprocal Ia inhibition, suggesting that exercise performance does not decrease during joint exercise.

At 30% MVC for the single condition and C–T intervals at 2 ms, the Sol H-reflex amplitude did not differ significantly. This result suggests that reciprocal Ia inhibition decreases in the Sol muscle depending on its contraction intensity. Excessive simultaneous contraction intensity also decreases joint movement (Hayashi et al., 1988; Kagamihara et al., 1993; Kagamihara and Tanaka, 1996). The intensity of co-contraction at the same contraction intensity was set to 30% MVC, which was the maximum intensity without joint movement; thus, maximum co-contraction intensity was fixed in our pilot study. Co-contraction helps to fix the ankle joint as the intensity of co-contraction increases (Nielsen et al., 1994). Therefore, reciprocal Ia inhibition of the antagonist was inhibited at 30% MVC with co-contractile intensity, which may have worked to allow contraction between antagonists.

The H-reflex amplitude was significantly decreased at 30% MVC compared with that at rest at C–T intervals of 4 and 5 ms. Previous studies have reported the involvement of D1 inhibition in the inhibition mechanism at C–T intervals of 5 ms or longer (Mizuno et al., 1971; Tanaka, 1974). D1 inhibition reportedly works at C–T intervals of 5–80 ms, with maximum inhibition at 20 ms (Mizuno et al., 1971; Tanaka, 1974). D1 inhibition is the inhibition of long-latency spinal reciprocal inhibition functions. The mechanism of D1 inhibition is believed to be multi-synaptic inhibition with group II fibers as centripetal inputs (El-Tohamy and Sedgwick, 1983), whereas the condition stimulation that induces D1 inhibition is believed to be presynaptic inhibition at the terminal Ia. This is because it does not inhibit the exercise evoked potential due to cerebral magnetic stimulation (Faist et al., 1996). As per the literature to date, there has been no report on the effect of contraction intensity change and D1 inhibition during co-contraction; however, D1 inhibition is reported to work during ankle joint dorsiflexion, with the amount of D1 inhibition being the same as that at rest, irrespective of contraction intensity, and plantarflexion has the same D1 inhibition as that at dorsiflexion (El-Tohamy and Sedgwick, 1983; Morita et al., 2001). Focusing on the changes in contraction intensity, D1 inhibition decreases as plantarflexion torque increases, although the difference is reportedly not statistically significant (Morita et al., 2001). However, determining inhibition based only on the results of the C–T intervals of 5 ms is difficult. Future investigations are warranted at the C–T intervals of 20 ms to clarify the function of D1 inhibition during co-contraction.

One limitation of this investigation is that it studied reciprocal inhibition at a middle latency of 3 to 5-ms C–T intervals. In reciprocal inhibition, short latency is reciprocal Ia inhibition (2 ms) and long latency is D1 inhibition (5–80 ms) (Mizuno et al., 1971; Tanaka, 1974). The reported reciprocal inhibition at middle latency involved not two, but three synaptic reciprocal Ia inhibitions (Crone and Nielsen, 1989). However, the influence of changes in contraction intensity on reciprocal inhibition at middle latency has not been reported. Therefore, changes in the TA and Sol contraction intensity and the inhibition pathway should be investigated to clarify their influence on reciprocal inhibition during middle latency. Here, we examined reciprocal Ia inhibition during co-contraction, focusing on contraction intensity. However, we did not consider joint torque, which has been described in several reports (Nielsen et al., 1992; Crone and Nielsen, 1994; Oya and Cresswell, 2008; Magalhaes et al., 2015). The effect of reciprocal inhibition during co-contraction should be clarified using combined contraction intensity and joint torque. Additionally, reciprocal inhibition should be assessed during co-contraction. Another limitation of this investigation is that whether the muscle activity reached the peak at the time of MVC measurement remains unknown, especially regarding Sol. We fixed the thighs and feet tightly and took maximum care to make MVC possible. Furthermore, because it is Sol, the knee joint was performed in the flexion position to eliminate the influence of the gastrocnemius muscle.

Reciprocal Ia inhibition under changes in contraction intensity during co-contraction is yet to be fully elucidated. The present study confirmed reciprocal Ia inhibition at TA and Sol co-contraction at 5 and 15% MVC at the same contraction intensity. During co-contraction at 5 and 15% MVC, antagonist inhibition may occur during joint movement. However, co-contraction at 30% MVC decreased reciprocal Ia inhibition, suggesting that joint fixation could be affected. The influence of changes in co-contraction strength on reciprocal inhibition at intermediate latency requires further investigation, and suppression during co-contraction should be studied by combining contraction intensity with joint torque. Future studies should also investigate the influence of changes in co-contraction intensity on the reciprocal inhibition of middle latency, as well as reciprocal inhibition during co-contraction, by combining contraction intensity and joint torque.

## Author Contributions

RH designed the study and wrote the initial draft of the manuscript. ME, SK, and HO contributed to analysis and interpretation of data and assisted in the preparation of the manuscript. All other authors have contributed to data collection and interpretation and have critically reviewed the manuscript. All authors approved the final version of the manuscript and agreed to be accountable for all aspects of the work in ensuring that questions related to the accuracy or integrity of any part of the work are appropriately investigated and resolved.

## Conflict of Interest Statement

The authors declare that the research was conducted in the absence of any commercial or financial relationships that could be construed as a potential conflict of interest.
